# Resistance against Penetration of Electromagnetic Radiation for Ultra-light Cu/Ni-Coated Polyester Fibrous Materials

**DOI:** 10.3390/polym12092029

**Published:** 2020-09-05

**Authors:** Kai Yang, Aravin Prince Periyasamy, Mohanapriya Venkataraman, Jiri Militky, Dana Kremenakova, Josef Vecernik, Roman Pulíček

**Affiliations:** 1 Department of Material Engineering, Faculty of Textile Engineering, Technical University of Liberec, 461 17 Liberec, Czech Republic; aravin.prince@tul.cz (A.P.P.); mohanapriya.venkataraman@tul.cz (M.V.); Jiri.Militky@tul.cz (J.M.); dana.kremenakova@tul.cz (D.K.); 2 Vecernik s.r.o, 468 21 Alsovice, Czech Republic; jvecernik@seznam.cz; 3 Bochemie a.s., 735 81 Bohumín, Czech Republic; Roman.Puilcek@bochemie.cz

**Keywords:** electromagnetic shielding effectiveness, electroless plating, Cu/Ni deposition, UV protection, electrical resistance, thermal radiation resistance, water contact angle

## Abstract

Resistance against penetration of various rays including electromagnetic waves (EM), infrared rays (IR), and ultraviolet rays (UV) has been realized by using copper (Cu)-coated fabrics. However, the corrosion of the Cu on coated fabrics influenced the shielding effectiveness of the various rays. Besides, the metal-coated fabrics have high density and are unbreathable. This work aims to solve the problem by incorporating nickel (Ni) into the Cu coating on the ultra-light polyester fibrous materials (Milife^®^ composite nonwoven fabric—10 g/m^2^, abbreviation Milife) via electroless plating. The electromagnetic interference (EMI), IR test, ultraviolet protection factor (UPF), water contact angle, and air permeability of the Cu/Ni-coated Milife fabric were measured. All the samples were assumed as ultra-light and breathable by obtaining the similar fabric density (~10.57 g/m^2^) and large air permeability (600–1050 mm/s). The Cu/Ni deposition on the Milife fabrics only covered the fibers. The EM shielding effectiveness (*SE*) decreased from 26 to 20 dB, the IR reflectance (*R*_infrared_) decreased from 0.570 to 0.473 with increasing *w*_Ni_ from 0 to 19.5 wt %, while the *w*_Ni_ improved the UPF from 9 to 48. Besides, addition of Ni changed the Cu/Ni-coated Milife fabric from hydrophilicity to the hydrophobicity by observing WCA from 77.7° to 114°.

## 1. Introduction

Currently, electromagnetic pollution has increased rapidly because of the rapid growth of smartphone, wireless, and utilization of other electronic devices [1]. Generally, these equipment’s emits the electromagnetic energy with respect to the different frequency which causes serious concerns to the exposed human body [2]. For example, Singh et al. [3] revealed that a majority of the subjects who were residing near the mobile base station complain of sleep disturbances, headache, dizziness, irritability, concentration difficulties, and hypertension. Besides, the possibility of the leakage of personal information from electronic devices via the near field communication (NFC) technology also exist [4]. To protect electrical equipment and human body from these damages, electromagnetic interference (EMI) shielding provides a solution. Shielding of electromagnetic waves is here achieved by the absorption and reflection of electromagnetic (EM) radiation in the metal-coated fabrics and its shielding effectiveness (*SE*) is given to the evaluate the EMI level [5]. To achieve this goal, the metal-deposited fabrics with enhanced electromagnetic shielding are considered.

Generally, the conductive paints [6], sputter coating [7], and electroless plating [8] are the most common methods to prepare the metal-deposited fabrics. Especially, the electroless plating is characterized as its advantages include uniform and coherent metal deposition, good electrical conductivity, and efficient heat transfer. Mechanisms of electroless platting as autocatalytic deposition is a special variant of deposition by reduction where metal ions contained in the bath are reduced in the presence of a catalyst. As a result, metal deposition on the fabric was realized [9]. It was found that less water pollution happened by using the electroless plating and no electricity was used during the electroless plating, which reduced the substantial emission of carbon dioxide (CO_2_) [10,11]. 

Till now, various metal materials including the copper (Cu) [12,13], silver (Ag) [14], nickel (Ni) [15], and so on have been deposited on the fabrics via electroless plating method. Depending on the different metal materials, EMI shielding effectiveness (EM *SE*) in the range of 20 to 100 dB has been measured [16]. However, the major negative effect was sensitivity of some metals (Cu, Ag,…) used for EMI shielding purposes to corrosion under ambient conditions [12,14], while the Ni was resistant to corrosion. It was also found that the close atomic number of Cu (29) and Ni (28) supported a high compatibility to crystallize in whole range of each element portions. Besides. Cu is a redox active metal and has the ability to donate and accept electrons to shift between reduced (Cu^+^) and oxidized (Cu^2+^) states, while Ni is generally responsible for increase of corrosion resistance and decrease of thermal and electric conductivity. Therefore, it was feasible to incorporate the Ni with Cu or Ag coating on the fabric for the EMI shielding [17,18].

Besides, the metal-coated fabric had been proved to have the infrared (IR) rejection [8]. By taking the aforementioned studies into consideration, the ray resistance over the various wavelength could be proposed. However, the metal-coated fabric was considered to have the high fabric density. Although the large amount of the EM wave was scattered, the lower breathability and the heaviness limited the application of the metal-coated fabric in the microelectronic device or micro smart materials.

The porous light fabric is potential substrate for the microelectronic materials and for the incorporation of smart materials into textiles or fabrics. Generally, nanofibrous membranes as the ultra-light fibrous materials could be used [19]. However, the cost could be higher when compared with normal thin nonwoven fabric in reality. So, it is worth studying the resistance against penetration of electromagnetic radiation of the Ni-incorporated metal-coated porous light fabric and their EMI performance systematically. In our previous work, the polyester fabric (Milife^®^ composite nonwoven fabric—10 g/m^2^, abbreviation Milife) was selected as a Cu-deposition substrate and the EM *SE* of the Cu-coated Milife fabric was obtained larger than 25 dB [20]. 

In this work, the main aim was to incorporate the Ni into the Cu coating on the Milife fabric and the EM *SE* was studied by controlling the Ni content on the Cu-coated Milife fabric. Besides, the resistance of the Cu/Ni-coated Milife fabric against penetration of IR and ultraviolet (UV) was also investigated. 

## 2. Materials and Methods 

### 2.1. Materials

Milife fabric (10 g/m^2^), 100% polyester filament composite nonwovens combining machine direction and cross direction oriented nonwoven layers, was purchased from JX Nippon ANCI, Tokyo, Japan. Besides, all chemicals for the electroless plating were purchased from Sigma Aldrich, Prague, Czech Republic.

### 2.2. Preparation of Cu/Ni Coated Milife Fabric

The Cu/Ni-coated Milife fabrics were prepared by using the same steps as shown in previous work. The three steps are described as follows:

- Precleaning and surface structure opening: Pre-cleaning process on Milife fabric was carried out by using 2.5% non-ionic detergent (Noigen LV-4) with temperature of 40 °C. This process was kept at pH of 7 and duration was 20 min. The pre-cleaned fabric was rinsed with deionized water for the effective removal of residual surfactants. Surface structure opening was realized by hydrolytic treatment of the fabric in 1% sodium hydroxide (NaOH) at 70 °C for 5 min with subsequent rinsing in demineralized water.
- Surface activation process was carried out by using an activation solution (CATAPOSIT44, produced by Rohm and Hass Company, Amsterdam, Netherlands) at 45 °C for 5 min, following which the pretreated Milife fabric was immersed in 10% hydrochloric acid (HCl) for 1 min. The demineralized water was used to wash the activated fabric.
- Deposition of Cu/Ni on the Milife fabric during plating was realized in a mixture of copper (CuSO_4_·5H2O) and nickel sulphate (NiSO_4_·6H_2_O) in weight ratio of 1:1 in the presence of triethanolamine (TEA). After depositing the Milife fabric in the mixture, homogeneous generation and deposition of Cu-Ni alloy nanocrystals was performed by metering a solution of sodium borohydride (NaBH_4_) in volume ratio of 1:1 into this mixture without access of air at temperature 22 °C. Composition and concentration of the solutions were the same for all samples, following the ratio like 1. 3 g CuSO_4_·5H_2_O, 3 g NiSO_4_·6H_2_O, 14 g TEA, 0,5 g NaBH_4_, and 1 g NaOH per liter. The pH of the prepared solution was 9.5. After deposition, the 2× subsequent rinsing in demineralized water was done.

During the preparation process, all the chemicals were used for the measurement without further modification.

To observe the effect of Ni on the properties of Cu/Ni-coated Milife fabric, eight samples were prepared by controlling the Ni weight percentage in the electroless plating process from 0 to 25 wt % (details in Table 1). The Ni weight percentage was expressed in relation to the weight content of NiSO_4_·6H_2_O of the total weight content of CuSO_4_·5H_2_O and NiSO_4_·6H_2_O in the chemical plating bath. Concentration of Ni and Cu in chemical plating bath were directly evaluated analytically by standard atomic absorption spectroscopy (AAS) method. The AAS was also used for determination of content of Ni and Cu on the surface of platted fabrics after quantitative dissolution in 35 wt % nitric acid, and the results are given in Table 1. Besides, the pure Milife fabric was used as control sample and labeled as N0.

### 2.3. Tests and Methods

#### 2.3.1. Morphology and Structural Analysis

The surface morphology of Milife fabric with and without Cu/Ni coating was observed under the scanning electron microscopy (SEM) (VEGA TESCAN Inc., Licoln, NE, USA) at 20 kV. The cross-section and the surface of the Cu/Ni-coated fabric were tested. 

Air permeability of Milife fabric with and without Cu/Ni-coating was measured by the machine FX3300 under 100 Pa according to ISO 9237.

#### 2.3.2. Electromagnetic Shielding Effectiveness (EM *SE*)

Electromagnetic shielding effectiveness (EM *SE*) was analyzed by using a device described in Šafářová et al.’s work [21] in accordance with ASTM D4935-10. During the measurement, temperature was kept at 27 ± 1 °C and relative humidity was kept at 40 ± 1%. EM *SE* of Cu/Ni-coated sample was measured over frequency range of 30 MHz to 1.5 GHz and the results were expressed in decibels (dB). The set-up consisted of a sample holder with its input and output connected to the network analyzer. A shielding effectiveness test fixture (Electro-Metrics, Inc., Johnstown, NY, USA, model EM-2107A) was used to hold the sample. The design and dimension of sample holder followed the ASTM method mentioned above. Network analyzer Rohde & Schwarz ZN3 was used to generate and receive the electromagnetic signals. The standard mentioned above determined the electromagnetic shielding effectiveness of the fabric using the insertion-loss method. The set-up of the instrument was well explained in the previous research manuscripts from our laboratory [22,23]. The set-up consisting of the distribution of electrical and magnetic fields were in the coaxial transmission. Before EM *SE* measurements, the instrument was calibrated. Ten repetitive measurements were conducted, and statistical analysis was done. From the tests, the *SE* and reflection loss (*SE*_R_) were obtained.

(1) $$\;SE=SE_{A}+SE_{R}+SE_{M}$$

Here, *SE*—shielding effectiveness, $SE_{R}$*—*reflection loss, $SE_{A}$—absorption loss,$\;SE_{M}$—re-reflection correction factor. Normally, $SE_{M}$ can be neglected. So $SE_{A}$ can be calculated according to Equation (1).

According to the Equations (2) and (3), the transmittance (*T*_EM_) and reflectance (*R*_EM_) can be calculated. Absorptance (*A*_EM_) can be obtained by Equation (4). In addition, the EMI value at 1.5 GHz was used to calculate the reflection loss (*R*_EM_), transmission loss (*T*_EM_), effective absorbance (*A*_eff*,*EM_), and the emissivity (*E*_m,EM_) according to the Equations (4)–(7).

(2) $$SE=-10\log_{10}\left(T_{\mathrm{EM}}\right)$$

(3) $$SE_{R}=-10\log_{10}\left(1-R_{\mathrm{EM}}\right)$$

(4) $$1=A_{\mathrm{EM}}+R_{\mathrm{EM}}+T_{\mathrm{EM}}$$

(5) $$A_{\mathrm{eff},\mathrm{EM}}=\left[\left(1-R_{\mathrm{EM}}-T_{\mathrm{EM}}\right)/\left(1-R_{\mathrm{EM}}\right)\right]$$

(6) $$SE_{A\mathrm{eff},\mathrm{EM}}=10\log_{10}\left(1-A_{\mathrm{eff},\mathrm{EM}}\right)$$

(7) $$E_{m,\mathrm{EM}}=1-R_{\mathrm{EM}}$$

#### 2.3.3. Electrical Conductivity

**Figure 1:**
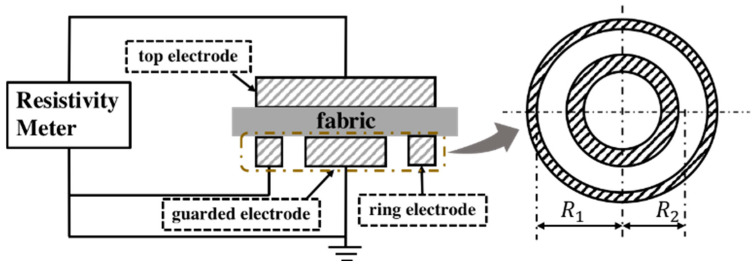
Measurement of electrical resistance for Cu/Ni-coated Milife fabric.

Surface resistance ($\rho_{s}$) of the Cu/Ni-coated samples was measured by using concentric electrodes (applied pressure 2.3 kPa) under a 100 V direct current (DC) power (Figure 1), in accordance with ATSM D257-07 standard. During the test, the electrodes were placed on the sample for 60 s and then surface resistivity values ($\rho_{s}$) were recorded. All samples were placed in the air-conditioned room 24 h prior to testing and the measurement was carried out with the temperature of 21 °C and the relative humidity (RH) was 45%.

#### 2.3.4. Spectral Reflectance Evaluation

**Figure 2:**
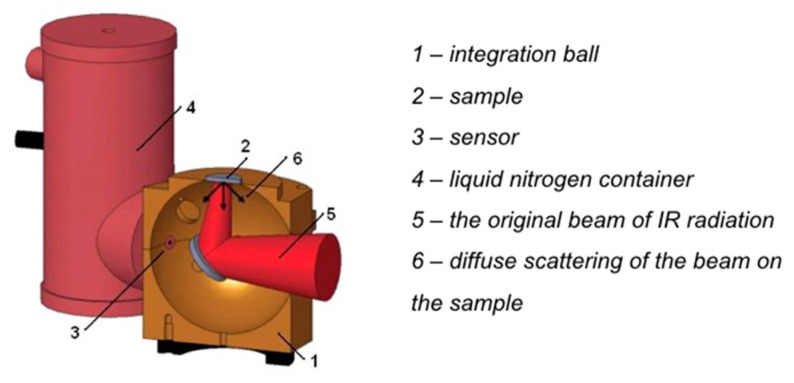
Scheme of the spectral reflectance measurement.

The spectral reflectance (*R*_infrared_) ranging from 2 to 20 μm (500–5000 cm^−1^) of textiles was tested by using the integration ball principle on the Mid-IR IntegratIR ™ (PIKE Technologies ™) (Figure 2). The beam stroked the specimen at an angle of 8° and interacted with its surface. The reflected radiation component was further diffusively reflected from the surface of the integration ball, and its value for individual wavelengths was recorded on a nitrogen-cooled measurement sensor. Besides, the spectral transmittance (*T*_infrared_) was also measured. Similar as the evaluation on the EM *SE* of the Cu/Ni-coated Milife fabrics (Section 2.3.2), the infrared curves of all the samples were calculated according to Equation (4), and the infrared absorbance (*A*_infrared_) was also obtained.

In addition, the IR values at 1000 cm^−1^ were focused. Both infrared emissitivty (*E*_m,infrared_) and infrared effective absorbance (*A*_eff,infrared_) were obtained as well by using the Equations (7) and (5) separately. Furthermore, the functional group of the Cu/Ni-coated Milife fabrics were characterized from the *A*_infrared_ and *T*_infrared_ curves. The temperature of the spectral measurement was kept 20 ± 1 °C by using the nitrogen (N_2_) gas.

**Figure 3:**
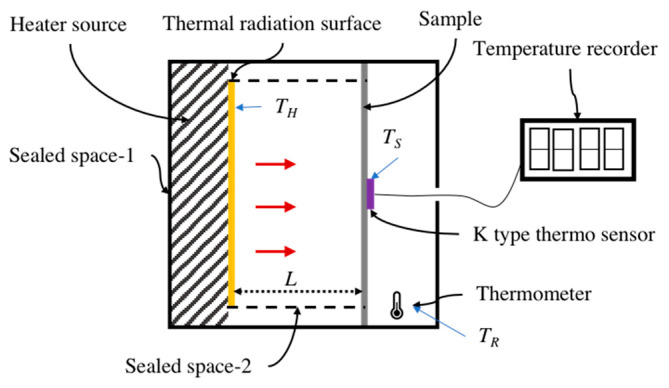
Measurement of thermal insulation for Cu/Ni-coated Milife fabric.

It was noticeable that the real thermal radiation reflection of the fabric was affected by the air property (speed, temperature, components,…). Especially, the porosity of the fabric also influences the thermal radiation reflection. Therefore, a custom-built set-up (Figure 3) was used to observe the temperature different between the heating source and the surface of the sample. The thin cotton fabric ($\epsilon_{\mathrm{rad}}$ = 0.95) attached on the heater source was used as thermal radiation surface and its temperature was set as 313.15 K (*T*_h_). The sample to be measured was placed at a distance 30 mm (*L*) from the thermal radiation surface. After 10 min, the temperature of the sample (*T_s_*) was recorded by K type thermo sensor connected with a temperature recorder (VOLFCRAFT IR 1201 50D). Besides, the thermal radiation surface and the measured sample were confined in a sealed space 1 ($\epsilon_{\mathrm{wall}1}$ = 0.95). To avoid the thermal convection, the sealed space 2 ($\epsilon_{\mathrm{wall}2}$ = 0.95) was set to enclose the aforementioned elements and its temperature (*T*_sur_) was around 298.15 K. Here, ϵ was the emissivity.

#### 2.3.5. Ultraviolet Protection 

The ultraviolet (UV) protection of the Cu/Ni-coated Milife fabrics was performed by using the Shimadzu UV 31001 PC ultraviolet-visiable (UV-Vis) Spectrophotometer with the spectral wavelength from 290 to 400 nm. The ultraviolet protection factor (UPF) value was calculated by using the following Equation (8).

(8) $$UPF=\frac{\sum_{290}^{400}S_{\lambda }E_{\lambda }\Delta_{\lambda }}{\sum_{290}^{400}S_{\lambda }E_{\lambda }T_{\lambda }\Delta_{\lambda }}$$

where, *S_λ_* is the irradiance of solar spectrum, *E_λ_* is the relative erythemal spectral response, *T_λ_* is the average spectral transmittance of the sample, and Δλ is the measured wavelength interval in nanometers.

#### 2.3.6. Water Contact Angle

Water contact angle for the Cu/Ni-coated Milife fabric was analyzed according to sessile drop principle by using the surface energy evaluation analyzer (Advex Instruments dependent on ISO: 27448:2009, Czech Republic). Deionized water was used for the contact angle measurement as a testing liquid. Total of 5 μL was used as the volume of testing liquid for all the measurement. In the measurement, the 5 μL of deionized water was dropped on the surface of Cu/Ni-coated Milife fabric by using micro syringe, and after 2 min the water contact angle was recorded. It was repeated ten times with different Cu/Ni-coated Milife fabric and the ten values were averaged. 

## 3. Results and Discussion

### 3.1. Morphology and Structural Analysis

**Figure 4:**
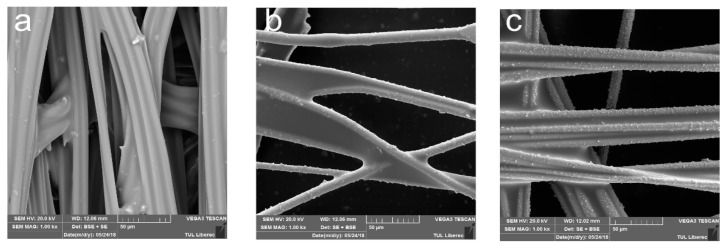
Microscopic images of Cu/Ni-coated Milife fabric (**a**) sample N0; (**b**) sample N5; (**c**) sample N8.

**Figure 5:**
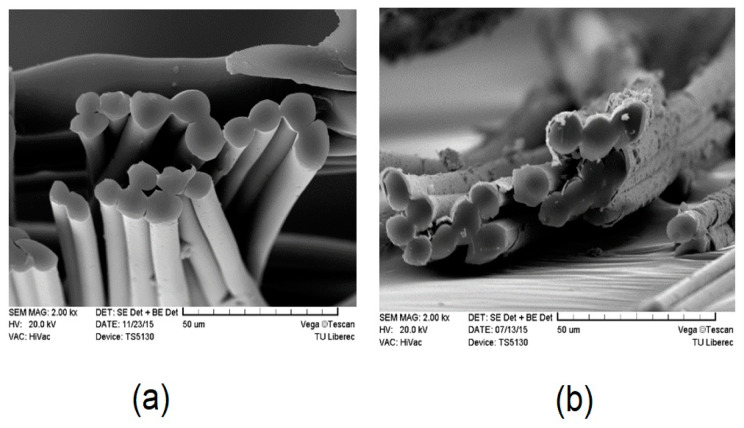
A cross sectional view of Cu/Ni-coated Milife fabric (**a**) sample N0; (**b**) sample N8.

The surface of Milife fabric with and without Cu/Ni-coating was observed from SEM images (Figure 4 and Figure 5). Control samples have plain surface without any metal addition, whereas the samples coated with Ni showed the deposition of metal particles on the surface. It was interesting to observe that the deposited Cu/Ni were on the surface of the fibers and did not fill in the interspace between the fibers significantly.

**Figure 6:**
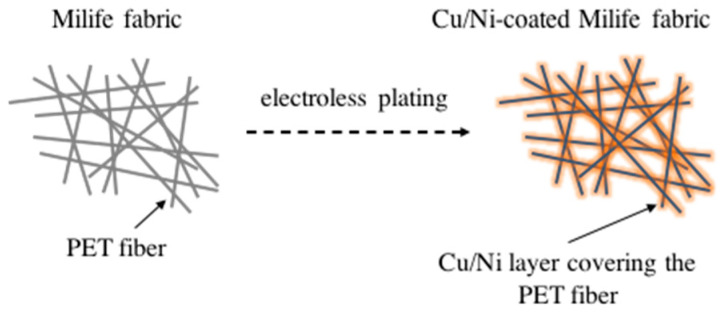
Structural change between the Milife fabric (N0) and the Cu/Ni-coated Milife fabric.

The structural change between the Milife fabric with and without Cu/Ni-coating is shown in Figure 6. It was found that Cu/Ni coating layer was very thin. It was also found that the Cu/Ni coating amount could be assumed as almost same (Table 1) and ranges from 0.47 to 0.60 g/m^2^. Therefore, the Cu/Ni-coated Milife fabric had the GSM ranging from 10.47 to 10.60 g/m^2^, which supported that the prepared samples were light.

**Figure 7:**
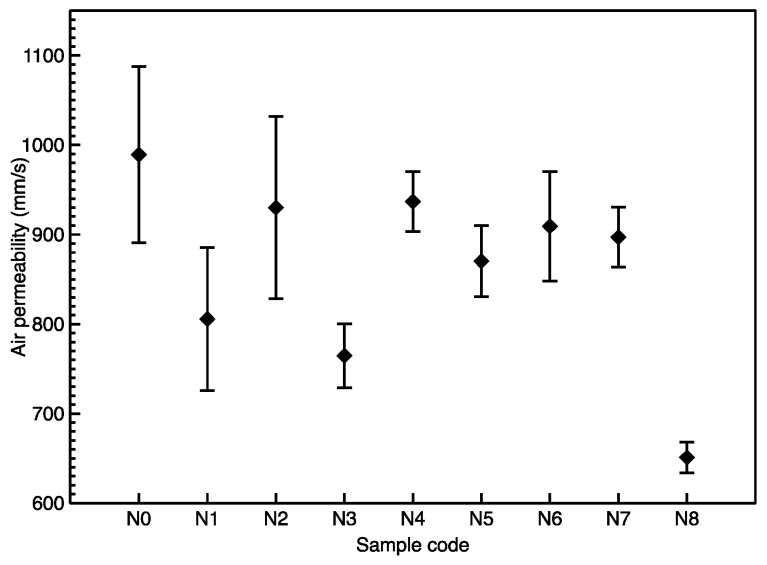
Air permeability of Cu/Ni-coated Milife fabric.

The air permeability of the Milife fabrics with Cu/Ni deposition (N1–N8) was slightly smaller than the Milife fabric (N0). There was no obvious difference in the air permeability between N1, N2, N3, N4, N5, N6, and N7 by observing the value ranging from 700 to 1000 mm/s, while the air permeability of the sample (N8) was significantly decreased to 600 mm/s (Figure 7). The difference may be caused by different Cu/Ni crystals on the surface of the fiber. Overall, the high air permeability (>600 mm/s) supported the breathability of the Cu/Ni-coated Milife fabric by comparing with other work [24,25].

### 3.2. Effect of Ni Content in the Cu/Ni-Coating Milife Fabric on EMI

**Figure 8:**
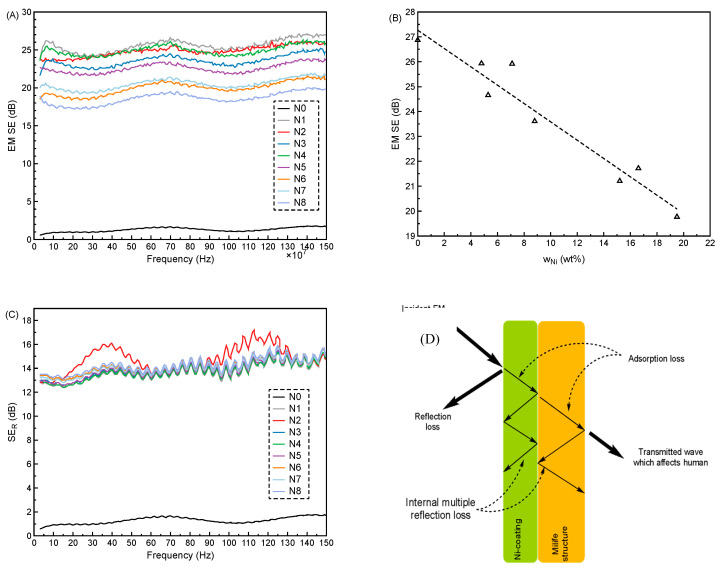

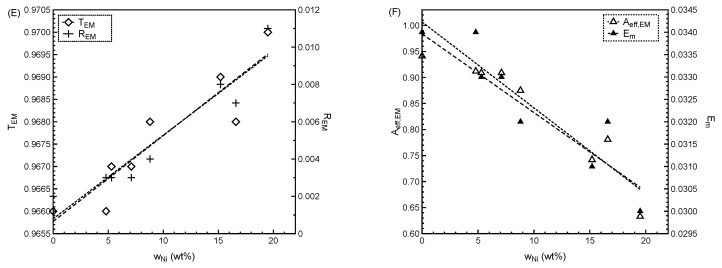
EMI results of Cu/Ni-coated Milife fabric (**A**) EM *SE* curves; (**B**) *SE*_R_ curves; (**C**) scheme of the EM in the fabric; (**D**) relationship between *w*_Ni_ and EM *SE;* (**E**) relationship between *w*_Ni_ and both *T*_EM_ and *R*_EM_; (**F**) relationship between *w*_Ni_ and both *A*_eff,EM_ and *E*_m,EM_.

EMI results including EM *SE* and *SE*_R_ with frequency ranging from 30 M–1.5 G are shown in Figure 8, and the evaluation of the EMI is calculated in the Table 2.

From the Figure 8A, it was found that the EM *SE* was obtained when there was Cu/Ni deposition on the Milife fabric since only the N0 (without Cu/Ni deposition) had the EM *SE* around 0 dB. The sample N1 (only with Cu deposition) was assumed the highest EM *SE* by observing the highest EM *SE* curve and the other samples N2–N8 (Cu/Ni-coated Milife fabrics) had the lower EM *SE* than the sample N1, which meant that the addition of Ni in the Cu coating on Milife fabric reduced the EM *SE*. The EM *SE* of the Cu/Ni-coated Milife fabric at 1.5 G decreased from 26.87 to 19.77 dB with the increasing w_Ni_ (Table 2). The linear relationship between the *w*_Ni_ and the EM *SE* was modeled in the Figure 8B, and *R*^2^ = 0.97. In addition, it was found that the effect of *w*_Ni_ on the *SE*_R_ was not as significant as on the EM *SE*, which was proved by the close *SE*_R_ curves shown in Figure 8C. The *SE*_R_ at 1.5 G of the Cu/Ni-coated Milife fabric increased from 14.67 to 15.22 dB with the increasing *w*_Ni_ (Table 2).

The trend of the EM *SE* and *SE_R_* of the Cu/Ni-coated Milife fabric over the *w*_Ni_ was different, which suggested the EMI mechanism was affected by the *w*_Ni_. It was well-known that the EM wave passing through the Cu/Ni-coated Milife fabric (Figure 8D) included the reflection *SE*_R_, adsorption *SE*_A_, and transmittance EM *SE*. To confirm the exact EMI mechanism in the Cu/Ni-coated Milife fabric, three parts including *SE*_R_, *SE*_A_, and EM *SE* at 1.5G were evaluated according to the Equation (2)–(4) and shown in Table 2. It was found that *R*_EM_ and *T*_EM_ increased while the *A*_eff,EM_ and *E*_m,EM_ decreased. The linear relationship between *R*_EM_, *T*_EM_, *A*_eff,EM_, *E*_m,EM_, and *w*_Ni_ was modeled separately in Figure 8E,F. The details of the relationship are shown in Table 3. The slope of the *A*_eff,EM_ was much higher than *R*_EM_ (0.0151 > 0.0002), which suggested that the *A*_eff,EM_ loss in the Cu/Ni-coated Milife fabric mainly accounted for the decrease of the EM *SE*. Namely, addition of Ni in the Cu coating on the Milife fabric increased the EMI reflection while seriously reduced the EMI adsorption. Besides, *E*_m,EM_ of the Cu/Ni-coated Milife fabric linearly decreased from 0.034 to 0.030 with the increased *w*_Ni_. 

Although the Ni had a negative effect on the EM *SE* of the Cu/Ni-coated Milife fabrics, the EM *SE* of the samples were above 20 dB except for the sample N8 whose EM *SE* was around 19 dB. According to the classification, the prepared Cu/Ni-coated Milife fabrics were evaluated in the “very good” category for general use (Table 4). 

Furthermore, the EM *SE* was in relation to the surface resistivity ($\rho_{s}$). As shown in Table 5, the $\rho_{s}$ of all the Cu/Ni-coated Milife fabrics (N1–N8) were smaller than 60 Ω/sq while the $\rho_{s}$ of the pure Milife fabric was as large as 13.45 MΩ. The Cu/Ni deposition gave the Milife fabric the good conductivity. Besides, no linear relationship between $\rho_{s}$ and *w*_Ni_ was found. The *w*_Ni_ did not change the surface electrical conductivity of the Cu/Ni coating on the Milife fabric.

### 3.3. UV Properties of Cu/Ni-Coated Fabric 

**Figure 9:**
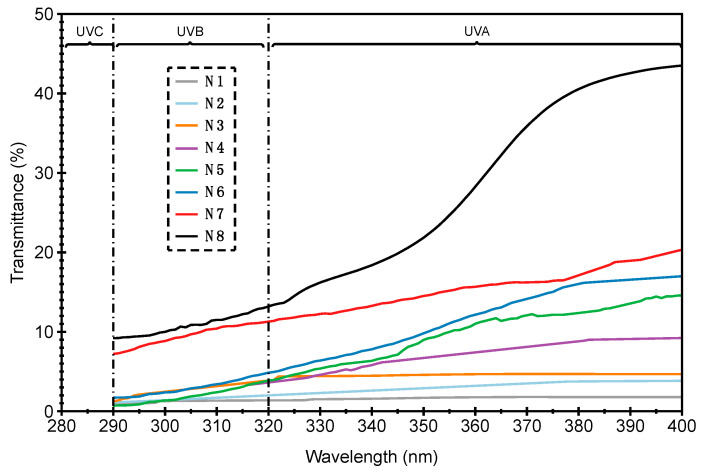
UV transmittance of Cu/Ni-coated Milife fabric over 290–400 nm (UVA and UVB).

The UV measurement of Cu/Ni-coated Milife fabric including both of ultraviolet radiation A (UVA) and ultraviolet radiation B (UVB) was shown in Figure 9. It was obvious that UV transmittance percentage of Cu/Ni-coated Milife fabric decreased with higher *w*_Ni_ over the UV electromagnetic spectrum. The UV transmittance percentage increased significantly in the fabrics N1, N2, N3, N4, and N5, and fabrics N6, N7, and N8 had less than 5% of transmittance all over the UV region (290–400 nm). The stable and good UV protective property was obtained when *w*_Ni_ was higher than 15.2 wt %. 

**Figure 10:**
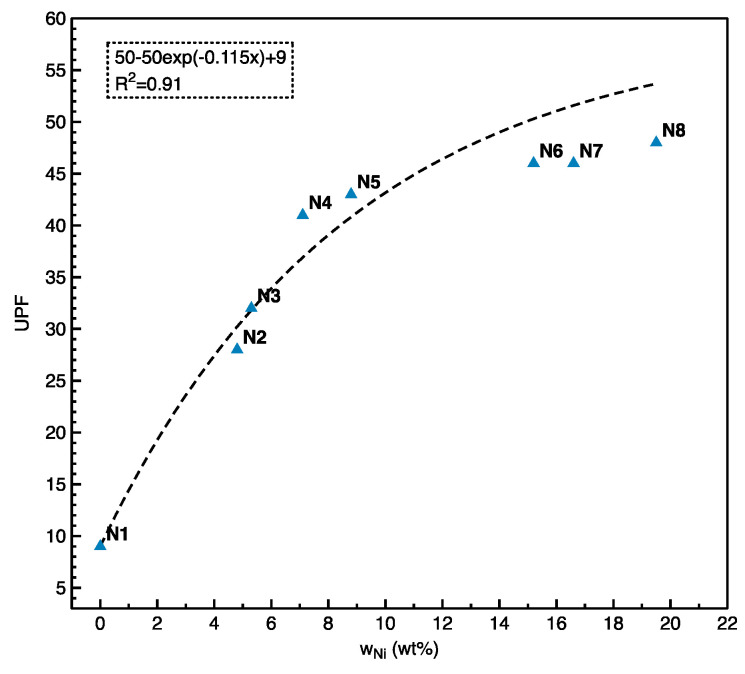
The relationship between UPF and *w*_Ni._

Figure 10 showed the UV protection capability of Cu/Ni-coated Milife fabric in terms of UPF values. As in sun protection factor (SPF) rating system used in case of sunscreens fabric, UPF rating is used to measure the UV protection [27]. Usually if the UPF value of fabric has equal or more than 50, it can provide the better protection by blocking the 98% of UV radiations. The Cu-coated Milife fabric (N1) had the lowest UPF value around 9, which was assumed to have little UV protective ability as their UPF values are about 8–10 (<15). When the coated fabric contained 2 wt % *w*_Ni_ (N2), the UPF value was 20.2. Similarly, the UPF was increased with the increased *w*_Ni_. On other hand, the fabric with higher *w*_Ni_ (>7.1 wt %) showed 40+ UPF ratings. The UPF values increased till *w*_Ni_ reached 15.2 wt % and thereafter it was saturated with no significant difference in the UPF values. It was found that the resistance against UV for the Cu/Ni-coated Milife fabric could be modified with increased *w*_Ni_ while the resistance against EMI was reduced with more *w*_Ni_. The reason may be that the Ni was able to dissipate the electromagnetic energy ranging from 290–400 nm. With respect to the *w*_Ni_ in the coated fabric, 16.6 wt % of Ni in the sample contributed to 46 UPF value and 19.2 wt % of Ni in the sample contributed to 48 UPF value. For statistical approach, we fitted the data between UPF value and *w*_Ni_ by using exponential function (Equation (9)), where *U*_f_ was set as 50 according to standard stating that excellent protection from UV when UPF was ≥50. *U*_i_ was the initial UPF of the Cu-coated Milife fabric (N1). *k_U_* was the estimated value giving the increasing rate of UPF with Ni deposition.

(9) $$y=U_{f}-U_{f}\exp\left(-k_{U}x\right)+U_{i}$$

*k_U_* was estimated as 0.115 and the final fitting model had *R*^2^ = 0.91, which confidently proved that there was an exponential relationship between *w*_Ni_ and UPF value.

### 3.4. Infrared Analysis of Cu/Ni-Coated Milife Fabric

**Figure 11:**
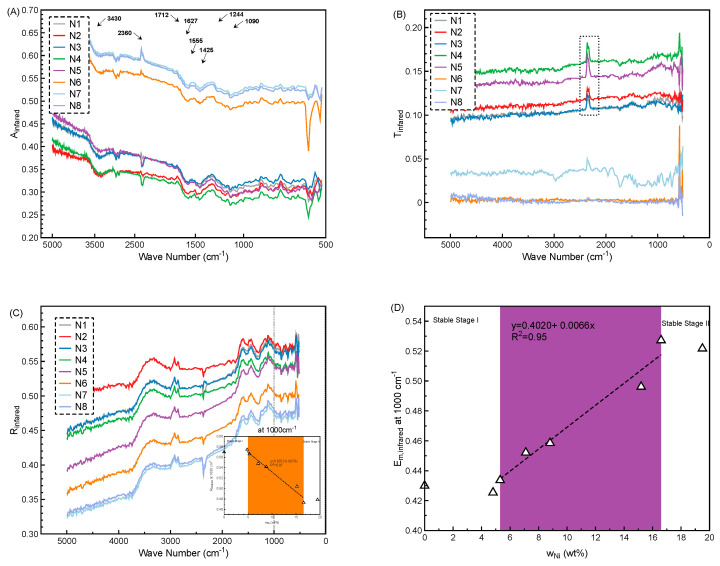
Analysis of infrared resistance of Cu/Ni-coated Milife Fabric (**A**) absorptance curves; (**B**) transmittance curves; (**C**) reflectance curves incorporated with the relationship between *w*_Ni_ and *R*_infrared_ at 10 μm; (**D**): relationship between *w*_Ni_ and *E*_m,infrared._

The infrared resistance evaluation on Cu/Ni-coated Milife fabric including reflectance (*R*_infrared_), transmittance (*T*_infared_) and adsorption (*A*_infrared_) was schemed in Figure 11 and the values at 1000 cm^−1^ are shown in Table 6.

*A*_infrared_ and *T*_infared_ curves were used to characterize the components of the Cu/Ni-coated Milife fabrics. It was found that all the samples had the same peaks in the *A*_infrared_ except for the peak ranging from 2360 to 2340 cm^−1^ (Figure 11A). The ester groups of all the samples were proved by observing the strong peaks at 1712, 1090, and 1244 cm^−1^ separately. The peaks in the range between 1712 to 1627 cm^−1^ and 1555 to 1425 cm^−1^ confirmed that there was a deposition of Cu and Ni particles on the surface of Milife fabric [20]. The peak at 3430 cm^−1^ represented the –OH group, which suggested that the hydrolysis and aminolysis happened during the electroless plating process and the strong interaction between metal particles and polyester was developed. However, the peaks of the samples ranging from 2360 to 2340 cm^−1^ were different, which represented the stretching and vibration of –C=N=O– or –N=C=O–. It was found that the peaks from 2360 to 2340 cm^−1^ became stronger with higher *w*_Ni_ (>4.8 wt %). It may be caused by the unstable coating process when the higher Ni molar was introduced in the electroless plating process, and the balance between the chemical reaction was affected. For the *T*_infared_ curves (Figure 11B), only the peaks ranging from 2360 to 2340 cm^−1^ were changed until the *w*_Ni_ was higher than 8.8 wt %.

*R*_infrared_ values were used to evaluate the resistance of the Cu/Ni-coated Milife fabric against penetration of IR. As seen in Figure 11C, all the samples had a similar *R*_infrared_ curve over the wave number from 516 to 5000 cm^−1^ by observing the same peak position and the similar increasing trend that *R*_infrared_ values increased with decreasing wave number and tended to be stable after the 1000 cm^−1^. The *R*_infrared_ at 1000 cm^−1^ of different samples were different which proved that the *w*_Ni_ affected the *R*_infrared_. By observing the *R*_infrared_ at 1000 cm^−1^ over the *w*_Ni_ (Figure 11C and Table 6), it was found that no obvious decrease of *R*_infrared_ happened until the *w*_Ni_ reached 5.5 wt %, and the *R*_infrared_ tended to be saturated when the *w*_Ni_ reached 16.6 wt %. Therefore, the influence of the *w*_Ni_ on the *R*_infrared_ was considered to be classified into two stages: the stable stage (I: *w*_Ni_ < 5.5 wt % and II: *w*_Ni_ > 16.6 wt %) and the negative stage (5.5 wt % < *w*_Ni_ < 16.6 wt %) (Figure 11C). On the one hand, the initial *R*_infrared_ was measured around 0.57 in the stable stage I and the lowest *R*_infrared_ was measured around 0.473 in the stable stage II. On the other hand, the negative linear relationship (*R*^2^ = 0.96) between the *R*_infrared_ and *w*_Ni_ was found in the negative stage. Similarly, the *E*_m,infrared_ values at 1000 cm^−1^ was calculated according to the Equation (7) and schemed in Figure 11D. The *E*_m,infrared_ values at 1000 cm^−1^ tended to be saturated to be around 0.527 when *w*_Ni_ reached 16.6 wt %. Although the addition of Ni had a negative effect on the resistance of the Cu/Ni-coated Milife fabric against penetration of IR, the highest *E*_m,infrared_ was around 0.527, which was still much smaller than the *E*_m,infrared_ of the normal fabric (*E*_m,infrared_ = 0.95 to 0.98). Therefore, the Cu/Ni-coated Milife fabrics had good resistance against penetration of IR.

Furthermore, the practical testing of the Cu/Ni-coated Milife fabrics for the thermal radiation resistance was carried out according to Section 2.3.4. The results are shown in Table 7. All the samples had the much lower *T*_s_ (302–304 K) than the *T*_h_ (313.15 K). In addition, a small decrease was observed by comparing the samples with Ni (N2–N8) with Cu-coated Milife fabric (N1), while there was no similar linear relationship between the *T*_s_ and *w*_Ni_, which was different from the aforementioned standard IR resistance analysis. The phenomena may be caused by the higher porosity of the samples, where the infrared transmittance and the unexpectable heat convection existed during the measurement. The change of the air components in the measurement also accounted for the difference as well.

Furthermore, by combining the results of the EMI and UV with the IR analysis, it was found that the addition of Ni only had a positive influence in the resistance against the penetration of electromagnetic waves when the wavelength only ranged from 290 to 400 nm in this case. Namely, the Ni had the selectivity on the resistance against the penetration of electromagnetic waves.

### 3.5. WCA on Ni-Coated Fabric

**Figure 12:**
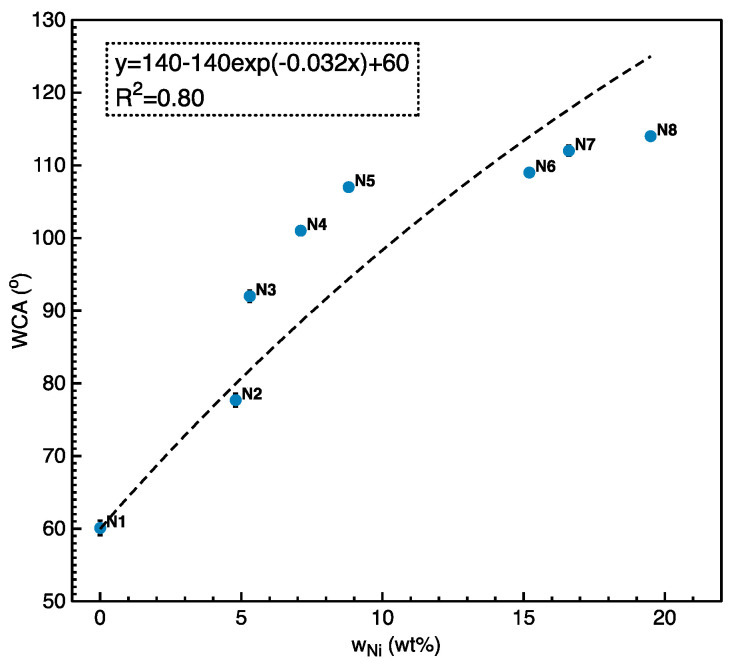
Relationship between WCA of Cu/Ni-coated fabrics and *w*_Ni_.

From the previous work, the WCA of the control sample (N0) was around 0°, while the Cu/Ni coating supported the WCA [28]. Figure 12 described the outcomes of the *w*_Ni_ on the wettability of the Cu/Ni-coated Milife fabrics. The Cu-coated Milife fabric (N2) had the WCA only of around 60.1 ± 1.0°. Meanwhile, the WCA of the Cu/Ni-coated Milife fabric increased from 77.7° to 114° with increasing *w*_Ni_. N7 and N8 had the WCA higher than 110° which confirmed the hydrophobicity of the surface. So, the erosion of the sample (N7 and N8) by the water could be relieved in reality [29].

In addition, the relationship between the WCA and *w*_Ni_ was roughly estimated by using exponential function (Equation (10)),

(10) $$y=C_{f}-C_{f}exp\left(-k_{C}x\right)+C_{i}$$

where *C*_f_ was set roughly as 140° which was the highest value of the pure Ni surface coating among the various studies [30], *C*_i_ was the initial WCA of the Cu-coated Milife fabric without any Ni deposition (N1), and *k_c_* [°/wt %] was the estimated increasing rate of WCA with Ni deposition. The *R*^2^ was 0.80 and *k_c_* was estimated as 0.032. 

## 4. Conclusion 

The present work successfully prepared the ultra-light Cu/Ni-coated Milife fabric (~10.57 g/m^2^). The Cu/Ni deposition only covered the fibers and the interspace between the fibers was observed. The lowest air permeability of the Cu/Ni-coated Milife fabric was larger than 600 mm/s, which supported the good breathability. The addition of Ni in the Cu coating on the Milife fabrics did not affect the interaction between the metal particles (Ni and Cu) and the Milife fabrics (polyester fiber) by observing same peak positions in the *A*_eff,infrared_ and *T*_infrared_ curves in the Section 3.4. The WCA was improved by increasing *w*_Ni_ and the Cu/Ni-coated Milife fabric became hydrophobic when the *w*_Ni_ reached 15.2 wt %, which extended the usage of the Cu/Ni-coated Milife fabric.

The EMI, IR, and UV results proved that the Ni in the Cu coating on the Milife fabrics had the selectivity on the resistance against the penetration of the electromagnetic waves over the different wavelengths, which could be controlled by the *w*_Ni_:

- Shielding EM wavelength. An obvious decrease in the EM *SE* of the coated Milife fabrics was observed when Ni was added. However, the EM *SE* of the samples were above 20 dB except for the sample N8 whose EM SE was around 19dB. A linear relationship between *w*_Ni_ and EM *SE* was found (*R*^2^ = 0.93). Besides, the $\rho_{s}$ of the Cu/Ni-coated Milife fabrics was smaller than 100 Ω/sq, which proved that the Cu/Ni-coated Milife fabrics had good conductivity.
- IR wavelength resistance. The standard measurement of the IR reflectance proved that the decrease from 0.570 to 0.473 in the *R*_infrared_ of the Cu/Ni-coated Milife fabrics was observed when the *w*_Ni_ increased from 5.5 to 16.6 wt %. However, the surface temperature of the Cu/Ni-coated Milife fabric was measured almost in the same way by the custom-built setup and 11 K less than the heating source. It could be suggested that the good IR resistance effectiveness was obtained by depositing Cu/Ni particles on the Milife fabric and Ni did not affect the IR reflectance significantly.
- UV wavelength resistance. It was interesting that the UPF was increased by adding the Ni in the Cu coating on the Milife fabric. The 40+ value was obtained when the *w*_Ni_ reached 15.2 wt %.

As a result, the sample N6 (*w*_Ni_) could be assumed as the optimized sample by considering the good shielding for EM, IR, and UV, but the Cu/Ni-coated Milife fabrics could also be selected for the specific usage. The prepared Cu/Ni could be used for the general applications like protection of the personal information from the near field communication, the protection of the pregnant woman from the electromagnetic radiation, incorporation with the outer wear for the protection from the UV and IR, and so on. Furthermore, it was interesting to observe that the effect of the increasing *w*_Ni_ on the ray resistance against the electromagnetic waves became positive with the lower wavelength especially when the wavelength became smaller than 400 nm. Such trend may initiate the development of the novel materials for the selective ray resistance.

## Figures and Tables

**Table 1:** Characteristics of the samples.

Fabric Code	${\mathrm{NiSO}}_{4}\cdot$6H_2_O Content in the Bath (wt %)	Total Mass of Cu/Ni on Surface (g/m^2^)	Ni Content in Cu/Ni Coating (*w*_Ni_) (wt %)
N1	0	0.572	0
N2	2	0.574	4.8
N3	4	0.571	5.3
N4	6	0.606	7.1
N5	8	0.577	8.8
N6	15	0.475	15.2
N7	20	0.517	16.6
N8	25	0.522	19.5

**Table 2:** Results of EMI evaluation at 1.5GHz frequency.

Sample Code	*SE* (dB)	*SE*_R_ (dB)	*SE_Aeff_* (dB)	*R*_EM_	*T*_EM_	*A*_eff,EM_	*E*_m,EM_
N0	0	0	0	0	0	0	0
N1	26.87	14.67	12.304	0.966	0.002	0.941	0.034
N2	25.93	14.70	10.544	0.966	0.003	0.912	0.034
N3	24.66	14.82	10.414	0.967	0.003	0.909	0.033
N4	25.92	14.80	10.414	0.967	0.003	0.909	0.033
N5	23.61	14.97	9.031	0.968	0.004	0.875	0.032
N6	21.21	15.08	5.882	0.969	0.008	0.742	0.031
N7	21.72	15.01	6.601	0.968	0.007	0.781	0.032
N8	19.77	15.22	4.357	0.970	0.011	0.633	0.03

*SE*: shielding effectiveness (dB), *R*_EM_: reflection loss, *T*_EM_: transmission loss, *A*_eff,EM_: effective absorbance, *SE*_R_: reflectance effectiveness (dB), *SE*_A_: absorbance effectiveness (dB), *E*_m,EM_: emissivity.

**Table 3:** Linear relationship between the EMI results and *w*_Ni._

Variations	Linear Relationship	*R^2^*
EM *SE* vs. *w*_Ni_	*y* = 27.2700 − 0.3680 *x*	0.93
*T*_EM_ vs. *w*_Ni_	*y* = 0.9657 + 0.0002 *x*	0.97
*R*_EM_ vs. *w*_Ni_	*y* = 0.0008 + 0.004 *x*	0.90
*A*_eff,EM_ vs. *w*_Ni_	*y* = 0.9839 − 0.0151 *x*	0.89
*E*_m,EM_ vs. *w*_Ni_	*y* = 0.0342 − 0.002 *x*	0.84

**Table 4:** Classification of EM *SE* values on textiles for general use [26].

Usage/ Grade	Excellent	Very Good	Good	Moderate	Fair
General use	*SE* > 30 dB	30 dB≥SE≥20 dB	20 dB≥SE≥10 dB	10 dB≥SE≥7 dB	7 dB≥SE≥5 dB

**Table 5:** Surface resistance of Cu/Ni-coated Milife fabric.

Sample Code	Surface Resistance (Ω/sq)
N0	13.45 ± 9.95 (× 10^7^)
N1	23.15 ± 11.95
N2	25.10 ± 3.54
N3	15.30 ± 0.71
N4	23.00 ± 2.40
N5	33.65 ± 6.01
N6	51.55 ± 4.74
N7	25.05 ± 0.64
N8	35.35 ± 1.34

**Table 6:** Evaluation of infrared resistance of Cu/Ni-coated Milife Fabric at 1000 (cm^−1^).

Sample Code	*R*_infrared_	*T*_infrared_	*A*_infrared_	*A*_eff,infrared_	*E*_m, infrared_
N1	0.570	0.118	0.311	0.726	0.430
N2	0.575	0.125	0.300	0.706	0.425
N3	0.566	0.115	0.319	0.735	0.434
N4	0.548	0.168	0.284	0.628	0.452
N5	0.541	0.155	0.304	0.662	0.459
N6	0.504	0.002	0.494	0.996	0.496
N7	0.473	0.030	0.527	1.000	0.527
N8	0.478	0.001	0.522	1.000	0.522

**Table 7:** Measurement of surface temperature of the Cu/Ni-coated Milife fabric under 313.15 K by Using Custom-built Setup.

Sample Code	*T*_s_ (K)
N0	304.25 ± 1.3
N1	302.45 ± 0.6
N2	302.25 ± 0.8
N3	302.65 ± 0.7
N4	302.05 ± 0.9
N5	302.15 ± 1.1
N6	302.55 ± 0.9
N7	303.15 ± 1.1
N8	302.05 ± 1.2
